# Reward and value coding by dopamine neurons in non-human primates

**DOI:** 10.1007/s00702-017-1793-9

**Published:** 2017-10-26

**Authors:** Aydin Alikaya, Mackenzie Rack-Wildner, William R. Stauffer

**Affiliations:** 1 Systems Neuroscience Institute, University of Pittsburgh, Pittsburgh, PA 15261 USA; 2 Department of Neurobiology, University of Pittsburgh, Pittsburgh, PA 15261 USA

**Keywords:** Dopamine, Learning, Decision making, Value, Monkey, NHP, Optogenetics, Reward prediction error

## Abstract

Rewards are fundamental to everyday life. They confer pleasure, support learning, and mediate decisions. Dopamine-releasing neurons in the midbrain are critical for reward processing. These neurons receive input from more than 30 brain areas and send widespread projections to the basal ganglia and frontal cortex. Their phasic responses are tuned to rewards. Specifically, dopamine signals code reward prediction error, the difference between received and predicted rewards. Decades of research in awake, behaving non-human primates (NHP), have shown the importance of these neural signals for learning and decision making. In this review, we will provide an overview of the bedrock findings that support the reward prediction error hypothesis and examine evidence that this signal plays a role in learning and decision making. In addition, we will highlight some of the conceptual challenges in dopamine neurophysiology and identify future areas of research to address these challenges. Keeping with the theme of this special issue, we will focus on the role of NHP studies in understanding dopamine neurophysiology and make the argument that primate models are essential to this line of research.

## Introduction

Rewards are a central feature of everyday life that promote learning, incentivize value-based decisions, and provide a currency for social exchanges. Dopamine neurons respond to rewarding events. Specifically, phasic dopamine activity reflects the difference between received and predicted rewards, i.e., reward prediction errors. Systems neuroscience studies in non-human primates (NHP) have been critical to understanding this coding scheme, as well as investigating the behavioral implications of reward prediction error coding.

Right now, we stand at the threshold of a new neuroscientific age. This age will be defined by big data and molecular control of neural information processing. From this vantage point, this review endeavors to provide a brief introduction to dopamine neurons, examine the critical findings revealed by NHP studies that have shaped our understanding of dopamine function, and evaluate what role this valuable species should have in future investigations. We will attempt to identify outstanding challenges to our current understanding of this critical brain system, propose areas for future research, and reinforce the need to preserve and technologically advance behavioral neurophysiology studies in NHP. Keeping with the theme of this special issue on NHP studies of basal ganglia function, this review does not attempt to be comprehensive and passes over many of the recent advances observed in rodent models. In addition, we do not discuss the role of dopamine cell loss in Parkinson’s disease, nor the valuable contribution of the monkey MPTP Parkinson’s disease model (Langston et al. 1983).

**Fig. 1:**
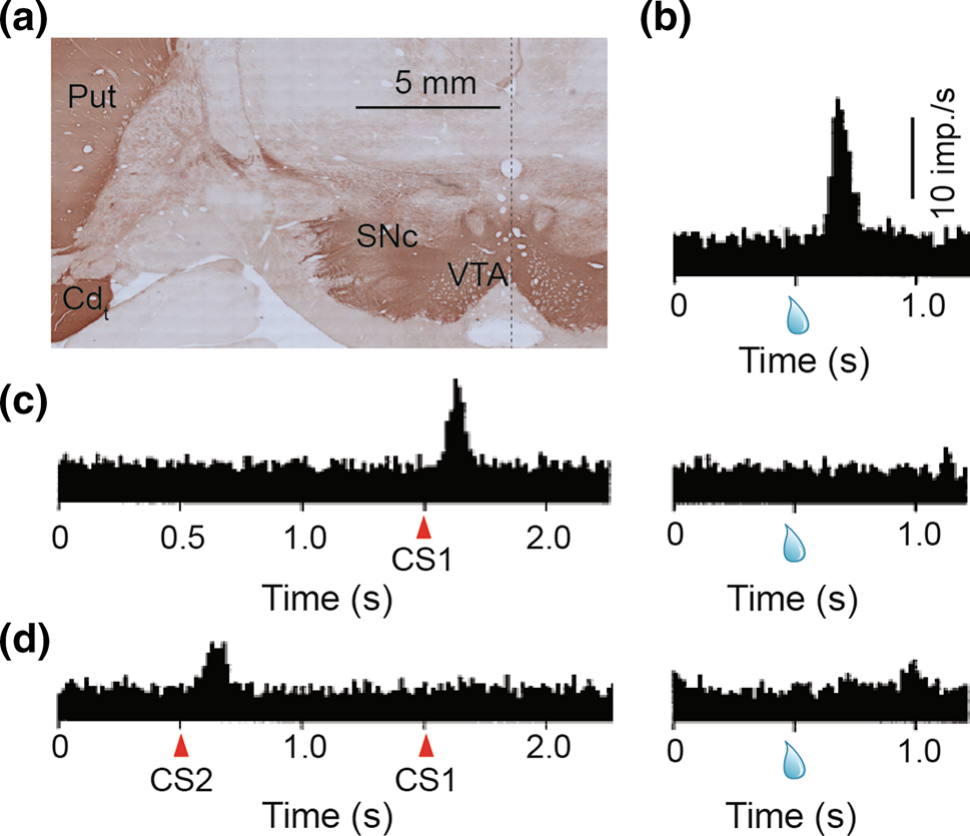
Dopamine anatomy and physiology. **a** Dopamine cell bodies in the VTA and SNc and dopamine terminals in the putamen and caudate tail are marked by brown DAB staining. *Cd*_*t*_ caudate tail, *Put* putamen, *SNc* substantia nigra pars compacta, *VTA* ventral tegmental area. **b**–**d** Dopamine responses code for reward prediction error. **b** Peri-stimulus time histogram (PSTH) of dopamine activity shows a strong response to unpredicted reward (indicated by the drop of juice). **c** PSTH of dopamine activity when a conditioned stimulus fully predicts reward. Dopamine neurons respond to the unpredictable onset of conditioned stimulus (CS1), but not to the fully predicted reward. **d** PSTH of dopamine activity when a high order conditioned stimulus (CS2) predicts the temporal onset of CS1 and delivery of reward. Dopamine neurons respond to unpredictable onset of CS2, but not to the fully predicted CS1 or reward. **b**–**d** Adapted from Schultz et al. (1993)

The majority of dopamine neuron cell bodies are located in the midbrain, in cell groups designated A8, A9, and A10 (Dahlstroem and Fuxe 1964). These groups broadly overlap with the retrorubral field (RRF), *substantia nigra pars compacta* (SNc), and ventral tegmental area (VTA), respectively. (Dahlstroem and Fuxe 1964; German and Manaye 1993) (Fig. 1a). Dopamine neurons express the enzyme Tyrosine Hydroxylase (TH) that converts the amino acid tyrosine to l-3,4-dihydroxyphenylalanine (l-DOPA). l-DOPA is converted to dopamine via aromatic l-amino acid decarboxylase (AAAD) and packaged into vesicles by the vesicular monoamine transporter (VMAT) (Lovenberg et al. 1962; Yelin and Schuldiner 1995).

Dopamine neurons are few in number, less than 200,000 in Rhesus macaque monkeys (Stark and Pakkenberg 2004). Despite this, dopamine terminals are found throughout the striatum (Lynd-Balta and Haber 1994a, b) (Fig. 1a) and primate frontal cortex (Smiley et al. 1994; Smith et al. 2014; Williams and Goldman-Rakic 1993, 1998). Dopamine neuron action potentials (AP) are recognized according to their distinct electrophysiological profile that features broad AP waveforms as well as low and irregular baseline impulse rates (Guyenet and Aghajanian 1978). Classical identification of dopamine neurons was based on the tight correlation between these distinctive waveform characteristics and apomorphine sensitivity (Bunney et al. 1973; Guyenet and Aghajanian 1978; Schultz 1986; Schultz and Romo 1987). A partial survey of the literature that used apomorphine injections to identify dopamine neurons revealed that 77 of 85 putative dopamine neurons were suppressed by apomorphine, whereas none of 39 putative non-dopamine neurons were inhibited by apomorphine (Aebischer and Schultz 1984; Bunney et al. 1973; Guyenet and Aghajanian 1978; Schultz 1986; Schultz and Romo 1987; Studer and Schultz 1987). New alternative techniques, such as optogenetic photo-identification and juxtacellular labeling, promise similar reliability (Brischoux et al. 2009; Cohen et al. 2012; Eshel et al. 2015; Stauffer et al. 2016; Ungless and Grace 2012). Photo-identification is especially promising, as it uses dopamine neuron selective expression of optogenetic channels coupled with optical stimulation to unambiguously identify many dopamine neurons in each animal (Cohen et al. 2012; Eshel et al. 2015; Lammel et al. 2012; Stauffer et al. 2016), but see (Lammel et al. 2015). This strategy promises a less-subjective criterion for dopamine neuron identification, compared to identification via waveform characteristics, and will be critical to achieve an unbiased picture of dopamine neuron diversity.

Dopamine neuron activity is traditionally divided into irregular, slow (0.3–8 imp/s) tonic activity and phasic (burst firing) activity when the impulse rate can briefly reach 20–30 imp/s (Grace and Bunney 1983). Phasic bursts of dopamine neurons are the most efficient way to change the dopamine concentration in their target structures (Gonon 1988). These phasic bursts are thought to respond to rewards, though there is significant disagreement about the degree of functional heterogeneity (see section “Challenges and future research directions”).

## Dopamine neurons code reward prediction error

Early evidence that dopamine neurons are involved in reward came from self-stimulation studies in maze-running rats (Olds and Milner 1954). However, behavioral neuroscience experiments in awake, head-fixed monkeys revealed the fundamental insight that phasic dopamine responses reflect reward prediction errors (Bayer and Glimcher 2005; Bayer et al. 2007; Bromberg-Martin et al. 2010; Enomoto et al. 2011; Fiorillo 2013; Fiorillo et al. 2003, 2008, 2013a, b; Hollerman and Schultz 1998; Kobayashi and Schultz 2008; Lak et al. 2014, 2016; Ljungberg et al. 1992; Matsumoto and Hikosaka 2009; Mirenowicz and Schultz 1994, 1996; Nakahara et al. 2004; Nomoto et al. 2010; Schultz et al. 1993, 1997; Stauffer et al. 2014; Tobler et al. 2005; Waelti et al. 2001). Reward prediction errors are defined in animal learning theory and machine learning as the differences between received and predicted rewards (Sutton and Barto 1998). A central tenet of animal learning theory is that temporal contiguity between a conditioned stimulus (CS) and reward (unconditioned stimulus, US) is not sufficient to drive learning. Rather, the reward must be unexpected; it must evoke a prediction error (Rescorla and Wagner 1972). Accordingly, reward prediction errors are teaching signals (Sutton and Barto 1998), and the reward prediction error nature of phasic dopamine responses strongly implicates these responses in learning.

Phasic dopamine responses are dependent on learning, and are time locked to unpredicted rewards and stimuli that elicit behavioral reactions (Mirenowicz and Schultz 1994, 1996; Romo and Schultz 1990; Schultz 1986; Schultz and Romo 1990). Early on in learning, when the associative strength between CS and reward is low, reward delivery strongly activates dopamine neurons (Ljungberg et al. 1992; Schultz et al. 1993) (Fig. 1b). Later, as the rewards become well predicted by the CS, dopamine neurons respond more strongly to the CS and less strongly to rewards (Ljungberg et al. 1992; Schultz et al. 1993) (Fig. 1c). With enough training, even higher order reward predictors (higher order CS) can activate dopamine neurons (Pan et al. 2005; Schultz et al. 1993) (Fig. 1d). Together, these studies provide overwhelming evidence that the phasic activity of dopamine neurons encodes reward prediction errors.

Dopamine reward prediction error responses are an ideal neural mechanism to mediate behavioral reinforcement learning, because they indicate both the occurrence of prediction errors and the proper direction to updating predictions. Rewards that are better than predicted activate dopamine neurons (positive prediction error responses), whereas rewards that are worse than predicted inhibit dopamine activity (negative prediction error responses). Modeling studies demonstrated that the prediction error term in popular reinforcement learning (RL) algorithms closely resembles the phasic dopamine signal (Montague et al. 1996; Schultz et al. 1997).

Using RL models, many studies have shown that dopamine responses conform to key principles of learning theory. For instance, when a US is predicted by a CS, a second CS presented at the same time or later than the first CS is ‘blocked’ from forming an association with the already predicted US, and dopamine neurons consistently fail to develop a response to the secondary, blocked CS (Steinberg et al. 2013; Waelti et al. 2001). Similarly, dopamine neurons are sensitive to temporal jittering of reward delivery. Early delivery of a predicted reward causes dopamine activation, whereas later than, predicted delivery leads to a diminished response (Hollerman and Schultz 1998). Moreover, dopamine responses reflect the discounting of future rewards, as suggested by reinforcement learning and economic theory (Enomoto et al. 2011; Fiorillo et al. 2008; Kobayashi and Schultz 2008). Trial-by-trial dopamine responses reflect the reinforcement history, a weighted average of past outcomes, in simple learning contexts (Bayer and Glimcher 2005). When the experimental context involves more complicated inter-trial task structure, dopamine neurons use this task structure to quickly update their responses on one trial, for instance, during reversal learning (Bromberg-Martin et al. 2010). Together, these results demonstrate the fidelity of dopamine responses to predictions made by learning theory, and they provide compelling evidence that phasic dopamine responses play a role in learning.

## Dopamine activity reflects economic value

The magnitudes of dopamine prediction error responses scale positively with reward parameters that increase value, including reward size (Bayer and Glimcher 2005; Tobler et al. 2005), and probability (Fiorillo et al. 2003; Lak et al. 2016; Nakahara et al. 2004; Nomoto et al. 2010), and negatively with reward parameters that decrease value, including delays (Fiorillo et al. 2008; Kobayashi and Schultz 2008) and bitter substances (Fiorillo et al. 2013b). Moreover, when monkeys indicate preference rankings between goods that have the same reward magnitude, expected value, and delay, dopamine responses vigorously reflect the preference rankings for reward type (Lak et al. 2014) and information content (Bromberg-Martin and Hikosaka 2009). These results indicate that dopamine reward prediction error responses reflect subjective value.

**Fig. 2:**
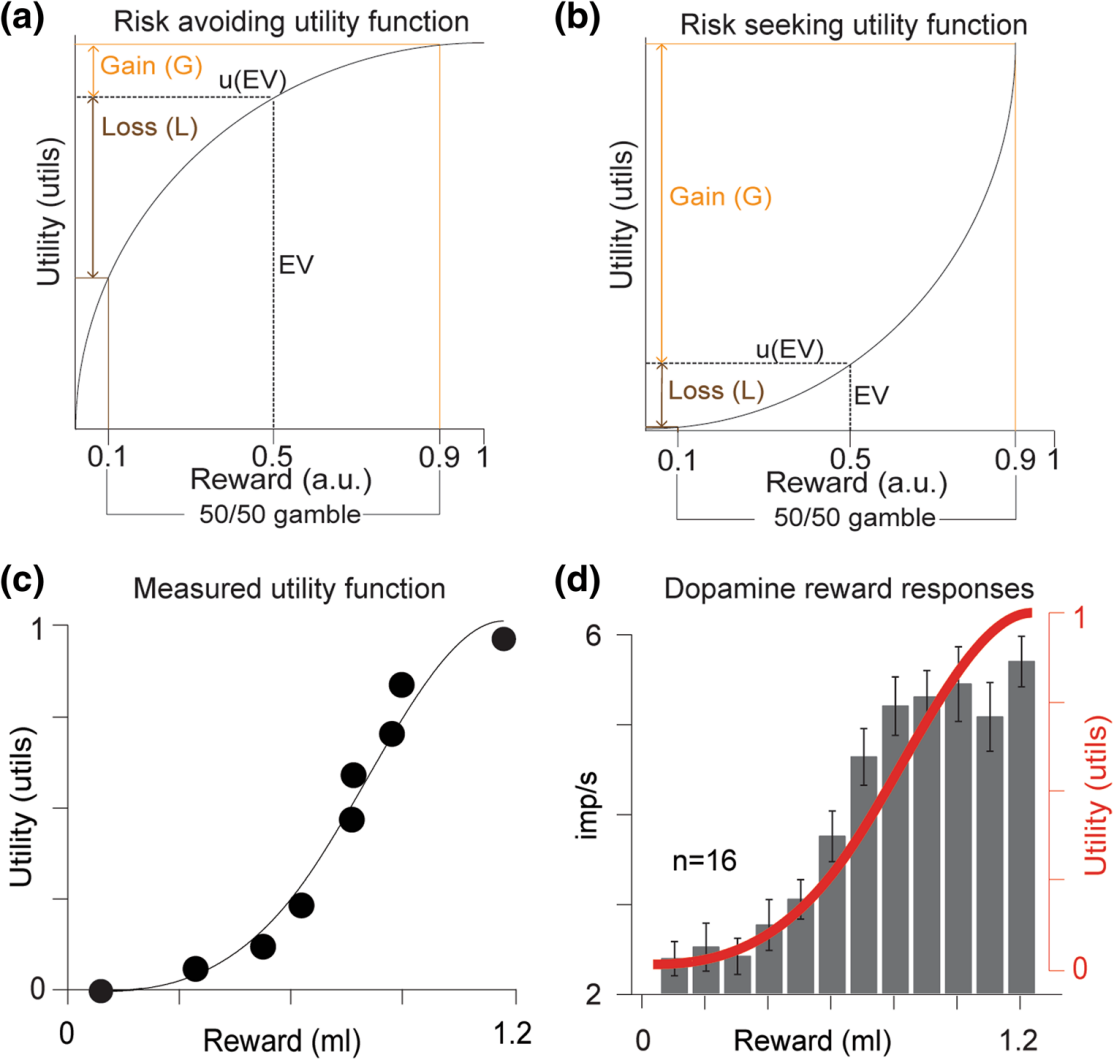
Phasic dopamine responses code value. **a**–**b** Example utility functions predict preferences between equi-probable (50:50) two outcome gambles (0.1, 0.9, arbitrary units) and the gambles’ expected values (EV) (0.5 a.u.). **a** Concave utility function indicates risk avoiding. **b** Convex utility function indicates risk seeking. Orange and brown two-sided arrows indicate the potential utility gain (G) and loss (L), respectively, relative to the utility of the expected value (uEV). For concave (risk avoiding) functions G < L, whereas for risk seeking (convex) functions G > L. **c** Measured utility function shows the utility of juice rewards. Convex regions of the utility (lower reward sizes) represent reward ranges, where the monkey was risk seeking. Concave regions (larger reward sizes) represent reward ranges, where the monkey was risk avoiding. Black dots represent points of subjective equivalence—termed certainty equivalents—between risky and safe rewards, measured through binary choices between risky and safe rewards. Solid line was fitted to the certainty equivalent data using cubic splines. **d** Dopamine neuron action potential responses are strongly correlated with the shape of the utility function. Action potentials were measured, while unpredicted rewards were delivered to the animals (sized 0.1–1.2 ml in 0.1 ml increments). Black bars represent impulse rate in a 500 ms window following reward. Error bars are SEM across 17 neurons. Red line represents utility functions and corresponds to secondary *y*-axis. **c**, **d** Adapted from Stauffer et al. (2014)

To demonstrate the functional relationship between subjective value and dopamine activity, it is necessary to measure a psychometric function of subjective value. Economic theory demonstrates that choices between risky options reveal subjective value (utility) as a function of physical value (Debreu 1959; von Neumann et al. 1944). Risk-avoiding individuals display concave utility functions, where the potential loss is greater than potential gain (Fig. 2a). In contrast, risk seekers have convex utility functions, where the potential utility gain outweighs the potential utility loss (Fig. 2b). A psychometric utility function with a consequential shape—a shape that can be meaningfully correlated with a neurometric function—can, therefore, be measured from choices under risk (Caraco et al. 1980; Machina 1987; Stauffer et al. 2014). Choices between risky rewards show that monkeys are risk seeking for small rewards (McCoy and Platt 2005; O’Neill and Schultz 2010; Yamada et al. 2013), but become more risk avoiding as rewards get larger (Genest et al. 2016; Stauffer et al. 2014). This behavioral pattern, risk seeking for small rewards and risk avoiding for large rewards, translates into a convex then concave utility function (Fig. 2c) (Genest et al. 2016; Stauffer et al. 2014). The magnitudes of dopamine responses to unpredicted rewards are highly correlated with the shapes of the measured utility functions (Fig. 2d). In addition, when more reward than delivered is predicted, the prediction error response of dopamine neurons depends on the local slope of the utility function (Stauffer et al. 2014). These results demonstrate that the fundamental variable coded by dopamine prediction error responses is the same variable used to make decisions.

An outstanding question related to economic value coding is whether economic costs reduce the magnitude of dopamine responses. In one study, increasing the effort required to get a reward resulted in reduced responses of some dopamine neurons, but not others (Pasquereau and Turner 2013). The behavioral measures used to gauge effort, however—reaction time and error rate—do not map linearly onto economic value. Thus, the true economic costs remained unknown. More studies are required to determine whether dopamine neurons code a net utility signal that accounts for the economic costs associated with effort.

**Fig. 3:**
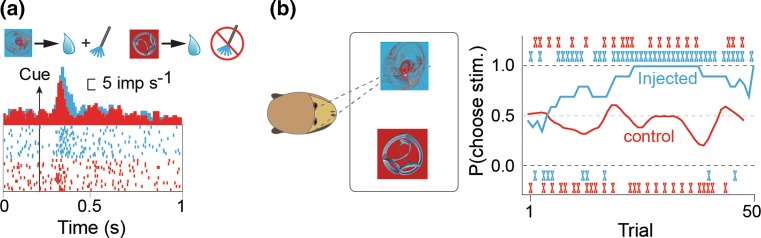
Optical stimulation of ChR2 expressing dopamine neurons leads to neuronal and behavioral correlates of value. **a** Top, monkeys viewed visual stimuli that predicted liquid reward delivered with (blue) or without (red) accompanying optical stimulation. **a** Bottom, larger neuronal response (blue) occurred to cues that predicted optical stimulation, compared to neuronal responses (red) to cues that did not predict optical stimulation. Blue raster plot and PSTH aligned onto the appearance of cues predicting reward plus optical stimulation. Red raster plot and PSTH aligned onto the appearance of cues predicting reward alone in the same neuron. **b** Monkeys made saccade guided choices between two visual cues (same reward scheme as in **a**). When the optical fiber was placed in the channelrhodopsin-infected hemisphere, monkeys learned to choose the cue that predicted optical stimulation, over the cue that did not predict optical stimulation (blue, ‘injected’). When the optical fiber was placed in the contralateral hemisphere, where no channelrhodopsin virus was injected, the monkeys continued to choose either option with equal frequency (red, ‘control). Thus, the monkeys’ choices indicated that optical stimulation added value. Two choice sessions are shown, one with the optical fiber in the infected hemisphere (blue) and one session with the optical fiber in the control, uninfected hemisphere (red). The ‘x’ indicates trial-by-trial choices in each session. The smoothed lines represent a running average of the choices (10 trial sliding window). This figure was adapted from Stauffer et al. (2016)

When behavioral decisions are made, dopamine responses reflect the chosen value, which is a post-decision variable (Lak et al. 2016; Morris et al. 2006). The current data suggest that dopamine neurons do not play a direct role in selecting options for a particular choice. Nevertheless, the close correlation between dopamine response to rewards and reward utility suggests that dopamine-teaching signals play a fundamental role in the choices we make over time. This functional role in value-based decisions was demonstrated by a recent study using optogenetic stimulation of dopamine neurons in a macaque monkey. Reward predicting objects that were followed by optogenetic activation of dopamine neurons were chosen more frequently than identical objects that were not followed by optogenetic activation (Stauffer et al. 2016) (Fig. 3). Thus, the likely role of the dopamine prediction error response is to train downstream brain structures about value.

## Challenges and future research directions

Despite the overwhelming evidence for the reward prediction error hypothesis of dopamine function, several outstanding challenges remain unaddressed. Here, we will attempt to define and outline three critical challenges deserving of attention. These include (1) determining whether dopamine neurons are functionally homogenous, (2) elucidating the relationship between behavioral measures and striatal dopamine release, and (3) defining the functional role of dopamine signals in the cortex.

### Functional diversity in dopamine neurons

Several studies have reported that a fraction of dopamine neurons are excited by novel or aversive stimuli and outcomes (Brischoux et al. 2009; Cohen et al. 2012; Fiorillo 2013; Fiorillo et al. 2013b; Lak et al. 2016; Matsumoto and Hikosaka 2009; Schultz and Romo 1987). Although there is significant anatomical diversity in the input–output pathways between medial and lateral dopamine neurons (Lynd-Balta and Haber 1994a, b, c; Watabe-Uchida et al. 2012), it remains unclear whether these non-reward-related activations represent the activity of distinct dopamine neuron circuits.

Multiple aspects of dopamine signaling may contribute to the observed complexity of dopamine responses, including context dependency and complex temporal dynamics. Dopamine neurons are exquisitely sensitive to the experimental context. For instance, when visual stimuli predict both appetitive and aversive stimuli, approximately 40% of dopamine neurons respond to the stimulus predicting the aversive outcome (Matsumoto and Hikosaka 2009; Mirenowicz and Schultz 1996). When the sensory stimuli are more perceptually distinct, as in when reward is predicted by an auditory cue and aversive outcome predicted by a visual cue, the number of dopamine neurons that respond to the aversive cue drops dramatically (Mirenowicz and Schultz 1996). This shows that stimulus context influences the activity of dopamine neurons. Likewise, the distribution of outcomes also alters dopamine responding. Highly rewarding contexts, such as behavioral situations with high reward probability, increase dopamine activations to neutral cues (Kobayashi and Schultz 2014; Matsumoto et al. 2016) and cues that predict aversive outcomes (Matsumoto et al. 2016). Even trial-by-trial behavioral measures in mice and monkeys predict whether dopamine neurons will respond to the current behavioral stimuli (Lak et al. 2017; Matsumoto et al. 2016). Such context-, and even trial-, specific effects seem to demand a systems neuroscience perspective that places focus on the behavior of the animal and not just the underlying neural circuits. Well-controlled experiments will be critical to understand the behavioral consequences of dopamine activity in real-world environments with complex emotional contexts.

The temporal dynamics of the dopamine response can further complicate the interpretation of these responses. Short latency activations can occur 50–90 ms following behavioral events that reflect physical impact, novelty, and stimulus generalization (Fiorillo 2013; Fiorillo et al. 2013b; Lak et al. 2014; Matsumoto and Hikosaka 2009; Nomoto et al. 2010). These short latency responses are not modulated by value, whereas later response components are (Fiorillo et al. 2013a; Lak et al. 2016). Likewise, robust rebound activations are often observed following negative prediction error responses when dopamine neurons can be silent for 200–500 ms (Bayer et al. 2007; Fiorillo et al. 2013b). Together, these complex dynamics, such as short latency and rebound activations, can complicate the interpretation of negative prediction error responses. It is important to note that an abundance of caution should be exercised with the interpretation of this neuronal behavior. Despite the various conclusions that can be reached by applying statistics to selected time windows, it is unclear how an aversive outcome-predicting stimulus that evokes a short latency activation, a long pause in firing, and then a rebound activation would influence dopamine release in the striatum.

### The relationship between behavior and striatal dopamine release

**Fig. 4:**
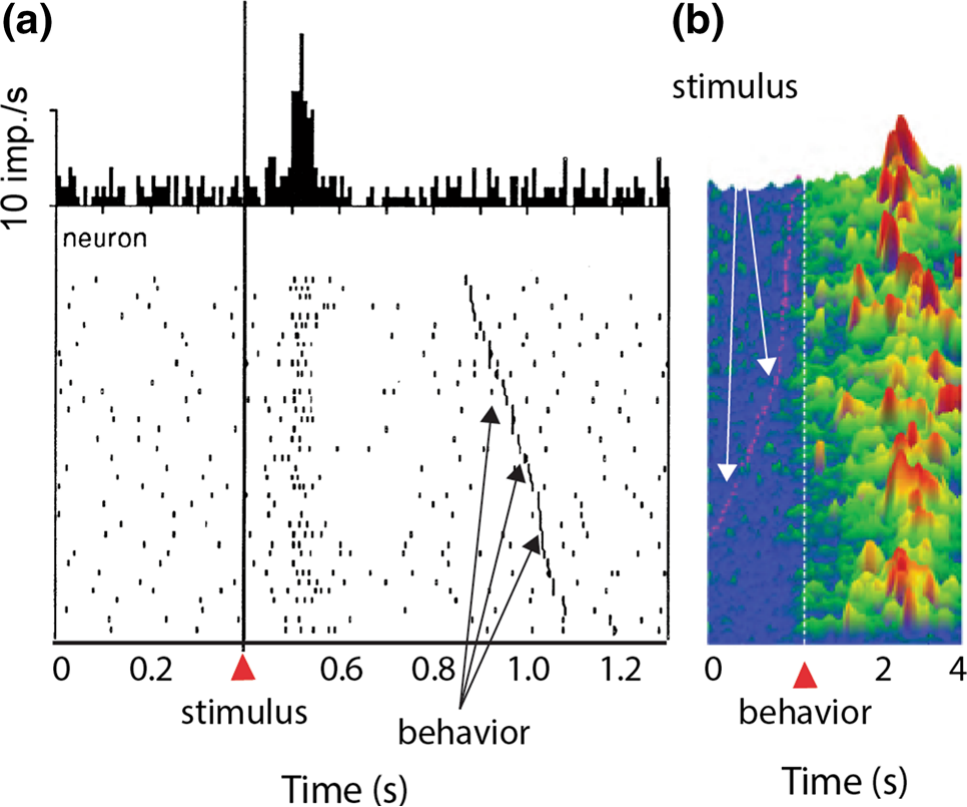
Temporal discrepancy between dopamine action potential responses recorded in the midbrain and dopamine release monitored in the striatum. **a** PSTH (top) and raster plot (bottom) of dopamine response to reward predicting cues. Responses were aligned onto cue onset (solid line). The time of movement onset during each trial is indicated by the dark hatches in the raster plot. This panel was adapted from (Schultz et al. 1993). **b** Profile of dopamine concentration change in the striatum of a rat after reward prediction. Dopamine concentration profiles are aligned to the time when the rats inserted their nose into a center port (white dashed lines). The time of instruction cues for each trial is indicated by the red ticks. This figure panel was adapted from Hamid et al. (2016)

Classic studies have repeatedly shown that, even during operant paradigms, dopamine responses are time-locked to external reward predictors, rather than to the onset of well controlled, single joint movements or associated EMG activity (Fig. 4a) (Ljungberg et al. 1992; Schultz et al. 1993; Schultz and Romo 1990). In contrast, larger, multi-muscle movements in monkeys (Schultz et al. 1983) or whole-body movements in rodents (Dodson et al. 2016; Howe and Dombeck 2016; Howe et al. 2013) are correlated with increased dopamine activity in midbrain cell bodies and striatal dopamine release sites. For instance, a recent study in freely behaving rodents found that phasic dopamine release was time-locked to behavior as well as stimuli (Fig. 2d) (Hamid et al. 2016). Investigating this discrepancy and exploring the larger question of how information processing in the striatum is modulated by incoming dopamine signals are of critical importance. Local striatal neurons (cholinergic interneurons) and afferent connections can influence dopamine release at dopamine terminals in the striatum (Cachope and Cheer 2014; Threlfell et al. 2012). This local influence might be especially significant in primates, because the basal ganglia are spatially organized according to cortical inputs (Alexander and DeLong 1985a, b; Alexander et al. 1986). The activity of dopamine neurons and cholinergic neurons is correlated in the NHP basal ganglia, but it is unclear how the behavioral variables coded by cholinergic neurons influence dopamine release (Morris et al. 2004). It is, therefore, important to characterize dopamine release in different functional regions of the striatum and observe the relationship between release and well-controlled behaviors. Recently, cyclic voltammetry was used to monitor dopamine reward responses in NHP striatum (Min et al. 2016; Schluter et al. 2014; Yoshimi et al. 2015), and this technique can shed light on how local network effects influence dopamine release and whether release reflects behavioral parameters other than reward, such as movements and actions.

### The role of fast dopamine signals in the frontal cortex

It is widely believed that phasic dopamine signals update action values in the striatum (Shen et al. 2008), but it is less well known what role these signals have in the frontal cortex. There was an extensive expansion of frontal cortex dopamine projections that accompanied the evolution of higher cognitive abilities in primates (Lewis et al. 1987; Smiley et al. 1994; Williams and Goldman-Rakic 1993, 1998). Accordingly, several studies have examined the role of dopamine in attention, working memory, and associative learning (Jacob et al. 2013, 2016; Noudoost and Moore 2011a; Puig and Miller 2012; Vijayraghavan et al. 2007; Williams and Goldman-Rakic 1995). These studies and others like them have relied upon the (relatively) slow process of agonist or antagonist infusion. Even when done with high spatial and temporal precision (Noudoost and Moore 2011b), these manipulations cannot approximate the natural dynamics of phasic dopamine signals. Instead, optogenetics can be employed for millisecond timescale control of dopamine release (Boyden et al. 2005; Tsai et al. 2009). Recently, a dual virus injection was shown to selectively label wild-type NHP dopamine neurons. Optical stimulation of cell bodies positively modulated behavioral read-outs of value. It was not known from that study whether the opsin was expressed in neuron terminals (Stauffer et al. 2016), but future research using next generation molecular tools in NHPs will permit projection specific recording and perturbation of neural activity.

## Conclusions

Electrophysiological recordings from dopamine cell bodies in the midbrain have demonstrated that phasic dopamine responses code reward prediction error, the difference between received and predicted reward. Studies in awake, behaving NHP have been critical to this endeavor, because they are highly trainable and can provide a wealth of data through in-depth exploration of single unit dopamine activity. NHP possess a rich and complex behavioral repertoire which has led to advanced understanding of the role of dopamine in learning, movement, and decision making. Not discussed here but worth mentioning, the MPTP monkey model has been critical to the study of neuronal and behavioral deficits associated with Parkinson’s disease. In short, the unique properties of NHP models have made them essential to understanding midbrain dopamine function and dysfunction.

The findings reviewed here demonstrate that, even for studying a relatively simple and evolutionarily old neural structure like dopamine neurons, there are significant advantages to using NHP models. Non-human primates possess behavioral and anatomical characteristics that are more similar to humans than any other experimental animal model. From a behavioral standpoint, the cognitive capability and choice flexibility reviewed here and demonstrated elsewhere (Eiselt and Nieder 2013; Stauffer et al. 2015) resembles human choice behavior. From an anatomical perspective, NHP dopamine projections to the striatum and frontal cortex are most analogous to those in humans. The NHP striatum contains the densest concentration of dopamine terminals (Lynd-Balta and Haber 1994a, b) and is functionally organized according to cortical inputs (Alexander and DeLong 1985a, b; Alexander et al. 1986). Likewise, the dopamine projections to the frontal cortex are massively expanded in NHP, where they primarily target executive and motor regions (Smiley et al. 1994; Williams and Goldman-Rakic 1993, 1998). For these reasons, and because of the clinical relevance of dopamine to numerous movement and mental health disorders including but not limited to Parkinson’s disease, dystonia, ADHD, OCD, psychosis, depression, and schizophrenia, it is critical to maintain and advance behavioral neurophysiology in awake, behaving primates.

A new generation of molecular tools—including optogenetics and in vivo single cell imaging—has revolutionized how we ask questions and even what questions we can ask. These technologies, however, have not been widely incorporated into monkey neurophysiology studies. Although progress is being made, as reviewed elsewhere in this issue (Galvan et al. 2017), there are many technical challenges impeding easy implementation of next generation molecular tools in NHP. Efficient light delivery, large-scale viral infection, and the lack of genetically modified NHP lines all pose significant challenges. Nevertheless, recent developments, including red shifted opsins and improved optical fibers (Acker et al. 2016), better recording/stimulating devices (Yazdan-Shahmorad et al. 2016), and virally mediated cell-type-specific ChR2 expression (El-Shamayleh et al. 2017; Klein et al. 2016; Stauffer et al. 2016), point towards a promising future for optogenetic NHP studies. These new technologies coupled with the enormous and oft-demonstrated utility of the NHP model should ensure continued focus on and research in this most evolutionarily relevant experimental species.
